# A heterozygous variant in *MEFV* in a familial autoinflammatory syndrome with PAPA-like features

**DOI:** 10.1186/1546-0096-13-S1-P68

**Published:** 2015-09-28

**Authors:** I Jéru, L Van Eyck, V Lagou, J Ruuth-Praz, B Copin, E Cochet, A Liston, A Goris, S Amselem, C Wouters

**Affiliations:** 1 Hôpital Armand Trousseau, Génétique et Embryologie Médicales, Paris, France; 2 KU Leuven, Microbiology and immunology, Leuven, Belgium; 3 KU Leuven, Neuroimmunology, Leuven, Belgium

## Introduction

Autoinflammatory disorders are a group of diseases whose nosology and etiology are only partly understood. Among Mendelian forms, familial Mediterranean fever (FMF), due to mutations in *MEFV*, is one of the most frequent. Most *MEFV* mutations are located in exon 10 and are usually associated with an autosomal recessive mode of inheritance. *MEFV* encodes pyrin, which interacts with PSTPIP1, a protein involved in the rare autosomal dominant pyogenic arthritis, pyoderma gangrenosum and acne (PAPA) syndrome.

## Objectives

We aimed to identify the underlying genetic defect in a very large family of Belgian ancestry with an autosomal dominant autoinflammatory syndrome showing PAPA-like features.

## Patients and methods

12 family members out of 22 spanning three generations presented with a PAPA-like syndrome. All patients suffered from childhood-onset recurrent episodes of fever, highly increased levels of acute-phase reactants, arthralgia, myalgia/myositis and neutrophilic dermatosis with variable manifestations (severe acne, skin abscesses, pyoderma gangrenosum, leucocytoclastic small vessel vasculitis). In between attacks low-grade systemic inflammation remained present. One patient also presented with cardiac failure which led to cardiac transplantation at the age of 18 years.

Linkage study was performed using 6K DNA chips. Whole-exome sequencing was carried out on two trios each consisting of an affected ‘child’ with an affected and unaffected ‘parent’. High throughput sequencing of the whole candidate region was performed in two patients using the SureSelect Custom Agilent library.

## Results

Through linkage analysis we identified a single 6.3Mb region on chromosome 16 segregating with the disease with a lod score of 3.6 and containing *MEFV*. Exome and targeted sequencing identified a single rare potentially functional sequence variantion in the linkage region. This variant is located in *MEFV* exon 2: c.726C>G; p.Ser242Arg, and was confirmed by Sanger sequencing. Whole-exome sequencing and high throughput sequencing of the target region did not reveal anyNo additional molecular defects, which might explain this autoinflammatory syndrome, were found by exome-sequencing of the target region.

## Conclusion

Our data reveal that a heterozygous variant in the exon 2 of *MEFV* could underlie an autosomal dominant autoinflammatory syndrome with neutrophilic dermatosis, as seen in PAPA syndrome. Consistent with this idea, heterozygous mutations in *MEFV* exon 2 were recently found in two patients who developed acute febrile neutrophilic dermatosis (Sweet syndrome) in the context of a myelodysplastic syndrome. Also, several autosomal dominant forms of FMF of smaller size have been reported previously, though the mutations were not located in exon 2. The present study underlines the close links between the nature of mutations in a given gene, the mode of inheritance, and the disease phenotype.

